# Advances with Lipid-Based Nanosystems for siRNA Delivery to Breast Cancers

**DOI:** 10.3390/ph16070970

**Published:** 2023-07-06

**Authors:** Md Abdus Subhan, Nina Filipczak, Vladimir P. Torchilin

**Affiliations:** 1 Department of Chemistry, ShahJalal University of Science and Technology, Sylhet 3114, Bangladesh; 2 Division of Nephrology, University of Rochester Medical Center, School of Medicine and Dentistry, 601 Elmwood Ave, Box 675, Rochester, NY 14642, USA; 3 Center for Pharmaceutical Biotechnology and Nanomedicine, Department of Pharmaceutical Sciences, Northeastern University, Boston, MA 02115, USA; 4 Department of Chemical Engineering, Northeastern University, Boston, MA 02115, USA

**Keywords:** lipid NPs, breast cancer, siRNA delivery, gene silencing, personalized therapy

## Abstract

Breast cancer is the most frequently diagnosed cancer among women. Breast cancer is also the key reason for worldwide cancer-related deaths among women. The application of small interfering RNA (siRNA)-based drugs to combat breast cancer requires effective gene silencing in tumor cells. To overcome the challenges of drug delivery to tumors, various nanosystems for siRNA delivery, including lipid-based nanoparticles that protect siRNA from degradation for delivery to cancer cells have been developed. These nanosystems have shown great potential for efficient and targeted siRNA delivery to breast cancer cells. Lipid-based nanosystems remain promising as siRNA drug delivery carriers for effective and safe cancer therapy including breast cancer. Lipid nanoparticles (LNPs) encapsulating siRNA enable efficient and specific silencing of oncogenes in breast tumors. This review discusses a variety of lipid-based nanosystems including cationic lipids, sterols, phospholipids, PEG-lipid conjugates, ionizable liposomes, exosomes for effective siRNA drug delivery to breast tumors, and the clinical translation of lipid-based siRNA nanosystems for solid tumors.

## 1. Introduction

**Figure 1:**
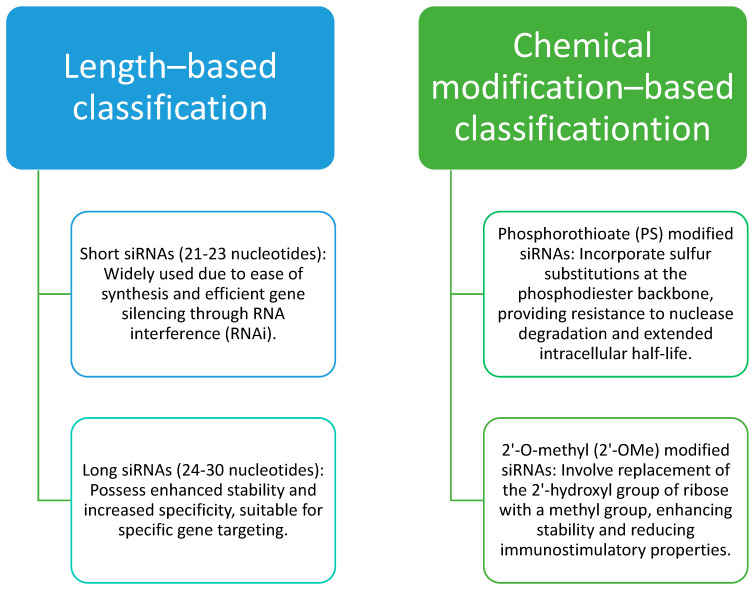
Classification of siRNAs used in breast cancer therapy [10].

Breast cancer is the most frequent type of cancer in women and is the main cause of cancer death, accounting for 2.08 million new patients and 0.67 million deaths every year [1]. Breast cancer has become the most prevalent form of cancer globally, surpassing lung cancer in 2020. It is also the most diagnosed cancer among women in the United States. It is a leading cause of cancer-related deaths in less developed nations and ranks as the second highest cause of cancer-related deaths in American women. In 2020, approximately 2.3 million women worldwide were diagnosed with breast cancer, resulting in 685,000 fatalities. Shockingly, every 14 s, a woman somewhere in the world receives a breast cancer diagnosis. Overall, breast cancer is the most frequently occurring cancer in women worldwide, being the top diagnosis in 140 out of 184 countries. It represents a quarter of all cancers affecting women globally. In the United States, breast cancer is the second most common cancer among women, following nonmelanoma skin cancer. In 2023, an estimated 300,590 people in the U.S. will receive a breast cancer diagnosis, with 297,790 of them being women, making it the most prevalent cancer among American women. Furthermore, in 2023, an estimated 2800 men will also be diagnosed with breast cancer in the U.S. [2]. Currently, breast cancer treatment is focused primarily on neoadjuvant or adjuvant therapy, surgery, and radiation therapy. Systemic therapy for breast cancer includes chemotherapy, hormonal therapy, targeted therapy, and immunotherapy. Breast cancer patient survival depends on the stage of disease, metastatic progression, and development of drug resistance [3,4,5]. Cancer cells demonstrate proliferative signaling, evasion of growth suppression, inhibition of programmed cell death, rapid angiogenesis, and stimulate invasion as well as metastasis. The cancer cells also can reprogram energy metabolism and evade immune responses. The reprogramming of energy metabolism and evasion of immune responses by cancer cells is enabled by genome instability and mutations. Thus, cancer qualifies as a heterogeneous disease. Breast cancers with their diverse diagnostic landscapes may influence clinical behavior and patient survival. Personalized therapies for breast cancers have been focused on targeting proteins and signaling pathways emphasizing the significance of novel molecular targets, utilization of efficient delivery tools, and early and accurate detection to enhance breast cancer therapy [1]. In the realm of breast cancer therapy, several specific small interfering RNA (siRNA) molecules have been investigated for their potential therapeutic applications. For instance, siRNA targeting the oncogene HER2 (human epidermal growth factor receptor 2) has been explored as a strategy to downregulate HER2 expression and inhibit the proliferation of HER2-positive breast cancer cells [6]. Additionally, siRNA targeting genes involved in cell cycle regulation, such as cyclin-dependent kinases (CDKs) and checkpoint proteins, have shown promise for the modulation of cell growth and induction of apoptosis in breast cancer cells [7,8]. Furthermore, siRNA molecules targeting angiogenesis-related factors, such as vascular endothelial growth factor (VEGF), have been investigated for inhibition of tumor vascularization to impair tumor growth in breast cancer [9]. siRNAs used in breast cancer therapy can be classified into different categories based on their structure and mode of action as shown in Figure 1. These examples illustrate the potential of siRNA-based therapies in breast cancer, providing targeted approaches to silence specific genes or pathways involved in cancer progression. 

Lipid-based nanosystems have served as promising siRNA drug delivery carriers for effective and safe cancer therapy [1]. SiRNAs are a small class of dsRNAs, consisting of 19 to 21 nucleotides. siRNAs impede gene expression and protein synthesis via complementary binding to their target mRNA, and they activate their target gene silencing through RNAi [11]. The potency of siRNAs may increase with length. A study demonstrated that a 27-nucleotide siRNA may be 100 times more potent than a conventional 21-neucleotide siRNA [12].

In recent years, lipidic nanosystems (Table 1) have emerged as promising vehicles for the delivery of small interfering RNA (siRNA). The physicochemical properties of lipidic systems play a crucial role in their effectiveness as carriers for siRNA delivery. One important property is the size and surface charge of the lipid nanoparticles. Generally, smaller nanoparticles have shown enhanced cellular uptake and improved penetration into tissues, making them desirable for efficient siRNA delivery [13]. Additionally, the surface charge of lipid nanoparticles influences their stability, interaction with biological barriers, and cellular internalization. Cationic lipids used in lipidic systems provide a positive charge, facilitating complex formation with negatively charged siRNA and promoting cellular uptake [14,15]. However, it is important to strike a balance, as excessive positive charge can lead to cytotoxicity and non-specific interactions. Hence, optimizing the surface charge of lipidic systems is crucial for achieving efficient and safe siRNA delivery. Another important physicochemical property is the lipid composition of the nanoparticle formulation. Lipidic systems for siRNA delivery often incorporate a mixture of lipids to modify desired properties. The selection of lipids influences the stability, encapsulation efficiency, and release profile of siRNA. For instance, the choice of lipids with appropriate hydrophobicity and chain length can improve the stability and loading capacity of siRNA in the lipidic system. Additionally, incorporating lipids with fusogenic properties can aid in endosomal escape, enabling effective siRNA release into the cytoplasm [16]. Furthermore, the inclusion of PEGylated lipids can enhance the stability and circulation time of lipid nanoparticles, reducing their clearance by the immune system [17]. Therefore, careful consideration of lipid composition is essential to optimize the physicochemical properties of lipidic systems for efficient siRNA delivery.

The first generation of lipid-based nanosystems employed cationic liposomes to encapsulate and protect siRNA molecules. Cationic liposomes possess a positive charge, enabling electrostatic interaction with the negatively charged siRNA, forming stable complexes that facilitate cellular uptake and endosomal escape. One such example is the widely utilized lipofectamine-based system, which combines cationic lipids with siRNA, leading to efficient gene silencing [26]. However, these early lipid-based nanosystems revealed challenges such as poor stability, limited siRNA loading capacity, and potential cytotoxicity.

To overcome these limitations, the second generation of lipidic nanosystems introduced the concept of lipid nanoparticles LNPs. LNPs are comprised of a lipid bilayer encapsulating the siRNA payload and often contain additional components, such as polyethylene glycol (PEG) and targeting ligands. These components enhance stability, circulation time, and specific delivery to the target cells or tissues. Notably, LNPs incorporating ionizable cationic lipids have shown remarkable success in siRNA delivery. For instance, the FDA-approved lipid nanoparticle system, Onpattro, utilizes an ionizable lipid formulation to effectively deliver siRNA targeting transthyretin (TTR) for the treatment of hereditary ATTR amyloidosis [27]. The second-generation lipidic nanosystems represent a significant advancement in siRNA delivery, addressing previous challenges and exhibiting improved therapeutic potential. Lipid nanoparticles act to reduce biological barriers to siRNA delivery, degradation by nuclease, and intracellular trafficking. LNPs may offer potential advantages including enhanced bioavailability, increasing aqueous solubility to reduce cellular clearance time, improving receptor specificity, and targeting drugs to exact tissue.

LNPs fabricated using well-tolerated components can be prepared on a large scale and can be sterilized and lyophilized, providing storage stability. They are also biocompatible and biodegradable, similarly to liposomes. The LNPs are composed mainly of phospholipids, organized in a bi-layered structure. LNPs form vesicles in the presence of water, increasing the solubility and stability of anticancer drugs when loaded into the carrier [28]. The LNP-based vehicles can encapsulate both soluble and insoluble drugs [29]. In addition to phospholipids, other compounds, including cholesterol, may be loaded onto the carriers. Cholesterol, in the LNP-based vehicles, reduces the membrane fluidity of the NPs, enhances its penetrability to insoluble drugs, and improves their stability in the circulation [28].

siRNA delivery of LNPs encapsulating siRNA facilitates effective and specific silencing of oncogenes, such as the tyrosine kinase receptors (TKRs) expressed on cancer cells since they are recognized as regulators of oncogenesis [30]. The most frequently utilized nanosystems for siRNA drug delivery include cationic, ionizable, neutral liposomes, exosomes, and other synthetic nanocarriers. These are simply prepared liposome-based delivery systems that have several benefits for in vitro and in vivo delivery of siRNA. 

The innate immune system provides a first line, and the adaptive immune system provides a second line, of the body’s defense [1]. The size of the LNP determines the Th1 response (IFNγ production) and Th2 response (IL-4 production). Particles of 40 to 50 nm improved Th1 stimulation, while larger-sized (>100 nm) particles activated the Th2 response [31]. The white blood cells and erythrocytes interact with LNPs depending on their various characteristics. These may affect the delivery of LNPs forming aggregates with the abundant erythrocytes, which may be toxic to immune cells [32]. The geometry of the LNPs may also influence the cellular internalization. The rod-like LNPs are internalized by the leukocytes with high efficiency, followed by spherical- and cylindrical-like NPs. However, cube-like LNPs are internalized less efficiently [32]. Cationic NPs can interact with cell membranes. However, cationic liposomes may increase immunotoxicity by activation of neutrophils and by prompting oxidative stress. Anionic LNPs have less favorable interactions with cell membranes due to repulsive forces and may exhibit poor cellular internalization [33]. Leukocytes-LNP interactions are beneficial because the resultant system crosses physiological barriers and moves into tumor niches [34].

Cationic liposomes are suitable for encapsulating therapeutic siRNA. The incorporated siRNAs within cationic liposomes demonstrate a superior uptake by the target cells through endocytosis. To target the desired cells effectively, ligand binding to the cell surface receptors of target cells was merged with the cationic coat of the liposome [35]. The fabrication of lipid-based nanocarriers has received much consideration due to the progress of systems for delivering siRNA to the cytoplasm of the tumor cells, protecting them from the circulatory environment that provides resistance to nucleases, overcoming immune responses enabling sufficient delivery to cancer cells [36].

The efficiency of LNPs for the delivery of siRNAs targeting CDK4 was evaluated in a study by Wang et al. [37]. The results of the study demonstrated that LNP-siRNA prompted efficient gene silencing compared to lipofectamine in MDA-MB-468-triple-negative breast cancer (TNBC) cells and triggered G_1_ phase cell cycle arrest. CDK4 inhibited cell cycle and proliferation in cancer cells. The advances in siRNA-based selective inhibiting agents targeting CDK4 may be an effective approach for breast cancer therapy. 

Lipid-coated calcium phosphate NPs loaded with siRNA promoted the siRNA delivery to TNBC cells. The NPs accumulated in tumor cells because of the enhanced permeability and retention (EPR) effect and the ability of siRNA drugs to target cancer cells. LNPs coated with calcium phosphate were utilized for the delivery of a mixture of siRNAs targeting genes required for cell survival [38]. The outcomes indicated an improved cellular uptake and hindrance of TNBC MDA-MB-468 cells. 

Effective delivery of chemotherapeutics and therapeutic siRNA has significant possible benefits. A co-delivery system consisting of paclitaxel (PTX) and siRNA coupled to LNPs significantly inhibited cancer cells utilizing targeted siRNA therapy [39]. Micellar LNPs carrying both PTX and siRNA targeting polo-like kinase 1 (PLK1) demonstrated synergistic tumor reduction in a xenograft murine model of MDA-MB-435s (a model cell line often used for the study of breast cancer) cells [40]. PLK1 is often overexpressed on breast cancer cell lines. Concurrent delivery of siRNA and chemotherapeutic drugs using cationic liposomes is an effective delivery system to target PLK1 [41,42]. The simultaneous delivery of PTX and PLK1 siRNA synergistically enhanced apoptotic MCF-7 cells and decreased angiogenesis. This combination delivery method demonstrated noteworthy benefits over single therapies using either PTX or siRNA [41]. Similarly, delivery of doxorubicin (Dox) with siRNA liposomes is a frequently used approach in cancer therapy [42].

The tumor inhibitory effect of siRNA loaded into liposomes targeting VEGF was effective against SKBR3 breast cancer cells compared to a commercial transfecting system, metafectene [43]. A polycation liposome encapsulating calcium phosphate (PLCP) NPs facilitated endosomal escape. The silencing function of PLCP/VEGF siRNA complexes was two times greater in MCF-7 cells compared to a commercial transfecting agent in an in vivo study using a xenograft model of MCF-7 cells. Additionally, a synergistic tumor hindrance effect was detected in tumor cells co-treated with doxorubicin in a mouse model. This study demonstrated that the PLCP/VEGF siRNA delivery system targeting VEGF is a promising approach to inhibit angiogenesis in breast tumors [44].

Ionizable liposomes with vincristine at a drug lipid ratio of 1:1 was tested in a breast cancer model [45]. In another study, ionizable liposomes conjugated to lipocalin 2(Lcn2) siRNA was used to inhibit the metastatic MDA-MB-436 and MDA-MB-231 breast cancer cell lines. The results indicated that the liposomal Lnc2 siRNA system can be effective in reducing breast cancer progression [46].

Cationic porphyrin lipid microbubbles loaded with hypoxia-inducible factor-1α siRNA along with photodynamic therapy were utilized for the treatment of TNBC, monitored using ultrasound imaging. HIF-1α siRNA down-regulated the HIF-1α expression, which was facilitated by the hypoxic tumor conditions or Ros generated during PDT. This approach improved PDT efficacy and reduced tumor development [47].

Lipid-coated calcium phosphate nanoparticles (LCP NPs) are a biocompatible, multifunctional efficient delivery system for cancer therapy. BsAb conjugated with LCP NPs targeted EGFR on MDA-MB-468 cells. These multifunctional LCP-BsA NPs were efficiently taken up by the tumor tissue. The integration of CD (cell death) siRNA and photothermal (ICG) therapy utilizing LCP-BsAb NPs hindered tumor growth in vivo in a mouse model [48,49].

The LNP surface was modified with HB-EGF antibody to target breast cancer [50,51]. HB-EGF is a ligand that binds to the EGF receptor (EGFR). TNBC tumors over-express HB-EGF [52]. Utilization of anti-HB-EGF antibody-modified LNP enhanced the siRNA delivery to breast tumors after intravenous injections. TNBC is a refractory disease with a poor prognosis. Nanomaterials such as functionalized mesoporous silica, chitosan-layered gold NPs, and cationic LNPs are utilized for siRNA delivery via an EPR effect [53,54,55]. However, active targeting may be utilized to deliver the LNPs containing siRNA to inhibit TNBC growth. TNBC growth was hindered by silencing PLK1 protein expression in tumors after intravenous injection of anti-HB-EGF antibody-modified LNPs incorporating siRNA against PLK1. This antibody alteration approach is an innovative strategy for the therapy of TNBC tumors [20].

Exosomes can be used as carriers for siRNA. Exosomes generated by the recipient’s own cells may be a prospective and safe carrier for siRNA delivery [56,57]. The surface of the exosomes can be modified with suitable ligands to improve the selectivity for target cells. Artificial exosomes or exosome mimetics may be advantageous for tissue-specific efficient delivery of siRNA [20]. Further, exosome-modified liposomes may be utilized for the targeted delivery of siRNA drugs for anticancer immunotherapy [58].

Major barriers to siRNA delivery that target an adequate dose to the tumor will enhance its internalization into tumor cells. Its release into the cytoplasm can be reduced with a complex comprising siRNA, cationic lipids, and pH-responsive peptide suitable for tumor uptake enhancement through focused ultrasound (FUS). The system offers efficient siRNA encapsulation, nuclease protection, endosomal escape, and effective gene silencing. Both lipid and ternary (lipid: peptide: siRNA complexes prepared with NIR fluorescently labeled siRNA) accumulate in tumors following FUS treatment. As a result, combining a well-designed lipid: peptide: siRNA complex with FUS therapy provides a prospective approach to achieve effective gene delivery in vivo and gene silencing [59].

Lyotropic mesophase LNPs consisting of an internal cubic phase nanostructure are known as cubosomes. In recent years, cubosomes have garnered attention as a promising drug delivery carrier for cancer therapy [60,61,62,63]. Cubosomes have many advantages over liposomes. Drug-loaded cubosomes can be utilized for cancer treatment. A cubosome functionalizing with HA to target CD44, which is highly expressed in TNBC cells, was utilized to incorporate siRNA to target breast cancer cells [62,63].

The siRNA drug was formulated with cationic lipid-assisted PEG-PLA NPs to target cyclin-dependent kinase 1 for TNBC therapy. The NPs inhibited c-myc overexpressing TNBC cells by reducing CDK-1 expression [64,65]. Further, aptamer-guided siRNA NPs targeting CD44 expression in TNBC were prepared [65]. The results of the study demonstrated that the drug exhibited improved anticancer effects against TNBC [65,66].

## 2. Recent Development of Lipid and Lipidoid-Based NPs for siRNA Delivery for Cancer Therapy

Advancements in understanding the molecular targets of cancer have opened the way for personalized therapy, which has overcome the complex and asymptomatic nature of various cancers. Targeted therapy, which interferes with specific molecular targets, has replaced the traditional approach of developing new chemotherapeutics. Over the past few decades, various molecules, such as tyrosine inhibitors, small molecule drug conjugates, serine/threonine inhibitors, and monoclonal antibodies, have been used to develop targeted anti-cancer therapies. Although these molecules have been effective, challenges related to protein stability remain. 

### 2.1. RNAi Therapy: Challenges and Advantages

**Figure 2:**
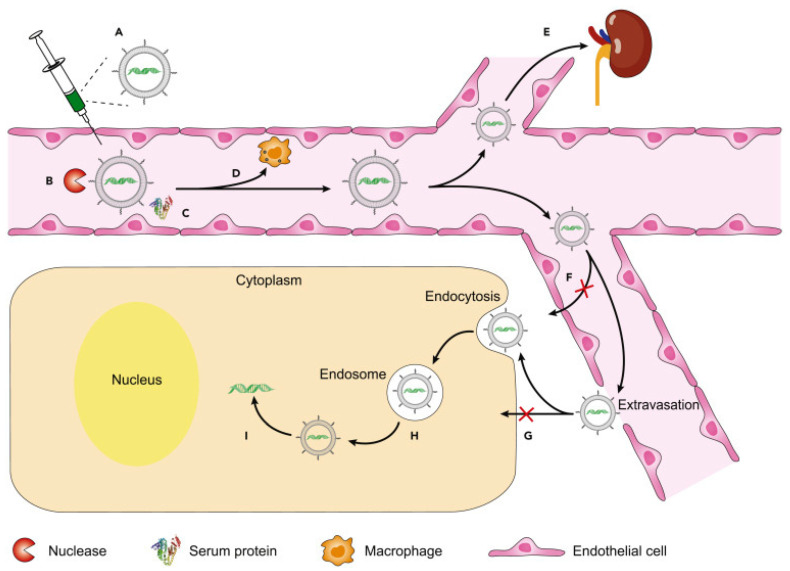
Challenges in in vivo delivery of RNA therapeutics using nanoparticles. (**A**) Spontaneous hydrolysis of RNAs. (**B**) Nuclease-mediated degradation. (**C**) Protein adsorption. (**D**) Immune recognition and clearance. (**E**) Renal clearance. (**F**) Endothelial barrier. (**G**) Cell membrane barrier. (**H**) Endosomal barrier. (**I**) Insufficient cargo release. Adapted from [69] with copyright permissions.

However, RNA interference has revolutionized targeted therapies by using non-coding RNAs to silence gene expression in various cells. Despite the existence of various options for gene silencing, such as TALENs and CRISPR/Cas, RNA interference remains the preferred option due to its precise mechanism, high potential, high specificity of gene silencing, and minimal off-target effects [67]. The main challenge associated with siRNA-based therapies for cancer and other diseases is delivering siRNA to the target cells in vivo in an effective manner. Successful use of siRNA-based drugs in the fight against cancer requires effective gene silencing in cancer cells. For effective siRNA delivery, the ideal systemic siRNA delivery system should possess several characteristics. These include biocompatibility, biodegradability, non-immunogenicity, protection of siRNA from serum nucleases during circulation and endosome release, avoidance of rapid hepatic or renal clearance, and promotion of siRNA delivery to target cells while avoiding normal tissues. Therefore, an in vivo, systemic siRNA delivery system should aim to increase the serum half-life of the siRNA, promote its distribution to target tissues, promote cellular uptake with intracytoplasmic release without degradation, and avoid off-target gene silencing activity. Several siRNA delivery systems have been developed for cancer therapy, including chemical modifications of siRNA, lipid-based delivery systems, polymer-based delivery systems, conjugate delivery systems, co-delivery of siRNA and anticancer drugs, and inorganic nanoparticles such as quantum dots, carbon nanotubes, and gold nanoparticles [68]. These modifications help address the problems associated with naked siRNA, such as serum stability, clearance of large molecular mass material, high toxicity, ligand-receptor interaction, vascular permeability to reach cancer tissues, and renal clearance as shown in Figure 2. 

However, achieving these benefits from a single modification is difficult, as siRNA delivery systems interact with various components. Poorly designed modifications can lead to problems such as a high positive charge on the surface of nanoparticles, which may cause unfavorable aggregation with erythrocytes [70,71]. 

### 2.2. Nanoparticles

RNAi-based cancer therapies have led to the use of nanoparticles as delivery molecules, which enhance transportation efficiency due to the EPR effect. However, preclinical characterization of the nanocarrier and studies on the release mechanism for loaded non-coding RNAs by these carriers must be analyzed before these molecules are taken into clinical trials [72]. 

Preclinical studies that evaluate the rate, range, and perpetuation of silencing as well as the timeframe of delivery of the carriers to the tumor site are crucial for improving siRNA delivery [70]. In recent years, there has been growing interest in the use of lipid and lipidoid-based nanoparticles (NPs) for the delivery of small interfering RNAs as a potential cancer therapy. siRNA is an encouraging therapeutic agent that can silence specific genes involved in cancer growth and progression, but its clinical utility has been limited by challenges in delivering it to cancer cells. Lipid and lipidoid-based NPs have emerged as an approach for delivering siRNA to cancer cells due to their biocompatibility, biodegradability, and ability to encapsulate and protect siRNA from degradation. Several lipid-based NPs have been developed for siRNA delivery in cancer therapy. Five major nanomaterial-based delivery platforms now include lipid, peptide, polymer, biomembrane, and inorganic NPs. Among various NPs, liposomes, exosomes, and lipid-like NPs are major categories that have attracted significant interest since the introduction of a cationic liposome formulated with 1,2-di-O-octadecyl-3-trimethylammonium propane (DOTMA) for therapeutic mRNA delivery into mammalian cells in 1989 [73]. Lipid-based drug delivery systems have several advantages including FDA approval, simplicity of the preparation, good biocompatibility, low immunogenicity, and high transfection efficiency. Lately, new ionizable lipids have been designed to replace the traditionally used cationic lipids to reduce toxicity without compromising transfection efficacy (Table 2). These ionizable lipids contain three moieties and have an optimal pKa value of 6.2–6.5, allowing for efficient encapsulation of RNAs into nanoparticles while maintaining neutrality at physiological pH and becoming protonated at lower cell pH to facilitate endosomal escape [74,75]. 

The choice of a lipid-based nanosystem for siRNA delivery depends on several factors, including the physicochemical properties of siRNA, the desired release kinetics, and the route of administration. The most natural and biocompatible vehicles that can be used for nucleic acid delivery are exosomes. 

#### 2.2.1. Exosomes

Exosomes are endogenous vesicles that have recently received much attention in the field of small interfering RNA delivery research. They are seen to be safe and efficient carriers. Exosomes used as siRNA carriers are preferably self-derived from exosome-producing cells to keep low immunogenicity. Although the use of exosomes as siRNA carriers is still under investigation, it has been reported that exosomes taken from human serum can be loaded with siRNA by electroporation and introduced into human lymphocytes and monocytes [76]. Similarly, liposomes are considered safe, biocompatible, and effective delivery systems for siRNA. They are spherical vesicles composed of phospholipids that can encapsulate siRNA.

#### 2.2.2. Liposomes

Liposomes also offer the possibility of co-delivering multiple drugs with different properties, such as hydrophilic and hydrophobic drugs, within the same particle. This can reduce the frequency of drug administration, which can improve patient compliance and reduce potential side effects [77,78]. Despite their advantages, liposomes also have some limitations, such as poor stability in biological fluids, susceptibility to opsonization by serum proteins, and potential toxicity associated with the use of certain lipid components. Thus, ongoing research continues to address these issues and explore new strategies to improve the clinical efficacy and safety of liposomal drug delivery systems [78,79]. 

#### 2.2.3. Solid Lipid Nanoparticles

Another commonly used delivery system is called the solid lipid nanoparticle (SLN). Those carrier systems are composed of natural lipids that are stabilized by surfactants in an aqueous solution, which distinguishes them from liposomes. SLNs are created using solid lipids such as glycerides, fatty acids, or waxes. By contrast, nanostructured lipid carriers (NLC) use a combination of solid and liquid lipids (oils) that form a solid blend at body temperature. The addition of oil prevents the formation of perfect lipid crystals, creating imperfections that increase the drug-loading capacity and physical stability of the lipid matrix [80,81]. Cationic compounds, typically lipids, are also used to let the negatively charged nucleic acids form a complex at the surface of the particle [82]. 

#### 2.2.4. Lipid-Polymer Nanoparticles

Another interesting delivery system uses lipid-polymer nanoparticles. LPNs are composed of a lipid bilayer and a polymer core that can enhance siRNA encapsulation and stability [83]. Lipidoid-based NPs are a newer class of NPs that have shown promise for siRNA delivery due to their ability to form stable complexes with siRNA molecules and efficiently deliver them to target cells. Lipidoids are modified lipids that can form NPs through self-assembly with siRNA. Lipidoid-based NPs have been shown to effectively deliver siRNA to cancer cells in preclinical models and have several advantages over traditional lipid-based NPs, including improved stability and reduced toxicity [83,84]. Despite the promising results of these preclinical studies, there are still challenges to be addressed in the development of lipid and lipidoid-based NPs for siRNA delivery for cancer therapy. These challenges include improving the stability and targeting efficiency of NPs, minimizing side effects, and optimizing the dosing and administration of NPs [20]. 

### 2.3. Targeted Therapy

The targeted delivery of drug nanoparticles to a specific site of action is crucial for effective treatment with minimal side effects. Passive targeting utilizes the enhanced permeation and retention effect, which occurs in many solid tumors due to leaky vascular blood vessels and incomplete lymphatic systems. Nanoparticles can accumulate in these incomplete vascular systems, and modifying their surfaces with hydrophilic macromolecules such as PEG can further optimize this effect. PEGylation can increase the stability of nanoparticles in biological fluids and reduce aggregation during storage and during in vivo application [85]. The main challenge is to design nanocarriers that can specifically recognize and target molecules presented on the tumor cell surface. Several different types of nanoparticles have been designed to meet this challenge, including nanoparticles that react to light, ultrasound, or heat to release the contents and disrupt cancer cells. Moreover, nanoparticles can be designed to carry multiple drugs simultaneously [86]. An alternate way to precisely transport the siRNA to the targeted site requires functionalization of the NPs’ surface by using various ligands. Consequently, structures presented on the cells’ surface or in tissues can be specifically targeted with proper ligands attached to the surface of the NPs [87]. The most used ligands include aptamers, small molecules, antibodies, peptides, polysaccharides, receptors, or antibody fragments. It has been proven that surface modification is a key factor affecting the binding and uptake of the nanoparticles once they reach the target site [88]. However, the effectiveness of active targeting is not so impressive, since studies have shown that only a small percentage of injected nanoparticles typically reach the tumor tissue. Despite this, surface functionalization can significantly influence the binding and uptake of nanoparticles by target tissues and can lead to improved therapeutic effects. It has been demonstrated that less than 1% of the administered nanoparticles can accumulate at the target site [89]. These findings imply that higher delivery efficiency is crucial for the achievement of lower NP dosages and reducing the cost of treatment where the current nanoparticles production cost is high. In addition, the remaining molecules that do not accumulate at the target tissue (~99%) are deposited in peripheral tissues, creating a risk of side effects. Finally, this very low delivery rate highlights an understanding that the exact delivery process is still unclear and further knowledge and research are needed to improve the delivery rate well above ~1% [89,90].

## 3. Promising Lipid-Based Nanosystems for siRNA Drug Delivery to Breast Cancer

Breast cancer is a complex and heterogeneous disease that poses significant challenges for treatment. Small interfering RNAs are a potent therapeutic approach that can silence specific genes involved in tumor growth and progression. However, the delivery of siRNAs to breast cancer cells remains a major obstacle. To overcome these challenges, researchers have developed various nanosystems for siRNA delivery, including lipid-based nanoparticles to protect it from degradation and deliver it to cancer cells. These nanosystems have shown great potential for efficient and targeted siRNA delivery to breast cancer cells [91,92].

### 3.1. Liposomes 

**Figure 3:**
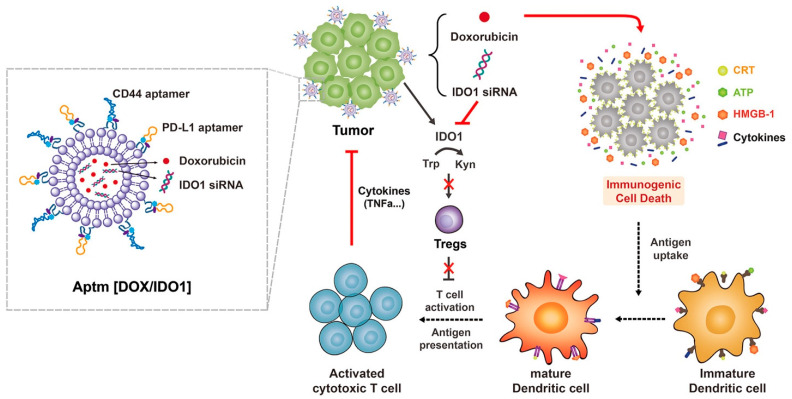
Aptamer-conjugated liposome for immunogenic chemotherapy with reversal of immunosuppression. Adapted from [99] with copyright permissions.

One promising lipid-based nanosystem for siRNA delivery to breast cancer uses liposomes. Cationic liposomes have a positive charge that can interact with the negatively charged siRNA molecules to form stable complexes. These complexes enter cancer cells through endocytosis and release siRNA into the cytoplasm, where it can silence specific genes involved in tumor growth and progression. The formulation of cationic liposomes affects their physical and chemical properties, which in turn affects their ability to form stable complexes with siRNA, protect siRNA from degradation in the blood stream, and deliver siRNA into target cells. Factors that can affect the formulation of cationic liposomes include the type and concentration of cationic lipid used, the presence of helper lipids, the ratio of cationic lipid to siRNA, the method of preparation, and the size and surface charge of the resulting liposomes [93]. Liposomes also offer an option of co-delivery of the anticancer drug and nucleic acid. Recently, paclitaxel (PTX) and siRNA (siPlk1) were co-encapsulated using cationic liposomes (CLs) to estimate the anticancer activity of the developed formulations using MDA-MB-231 and MCF-7 cell lines. The developed formulation showed sustained drug release for up to 168 h and significantly increased the biological half-life of PTX when compared to the marketed PTX formulation and enhanced its anticancer activity [94]. To counteract PTX resistance and enhance the anticancer effects of PTX, another nanosystem was created that consisted of a redox-sensitive cationic oligopeptide lipid (LHSSG2C14), a natural soybean phosphatidylcholine (SPC), and cholesterol. This liposome-based system, called PTX/siRNA/SS-L, delivers both PTX and anti-survivin siRNA, which specifically downregulates survivin overexpression. The increased expression of survivin in breast cancer cells is a significant contributor to the resistance of breast cancer cells to paclitaxel. The PTX/siRNA/SS-L system was found to have high encapsulation efficiency and released both PTX and siRNA quickly in response to redox changes. In vitro studies on 4T1 breast cancer cells showed that PTX/siRNA/SS-L exhibited elevated cellular uptake, endosomal escape, lower survivin expression, reduced cell viability and wound healing rate, and higher apoptosis rate. Moreover, in vivo experiments with 4T1 tumor-bearing mice demonstrated that PTX/siRNA/SS-L exhibited lower toxicity and inhibited tumor growth and pulmonary metastasis [95]. Another approach used to improve paclitaxel activity was to develop liposomes co-encapsulating paclitaxel, crizotinib (CRI), and Bcl-xL siRNA [96]. A different approach utilizing liposomes for targeted delivery of siRNAs into breast cancer has also been applied. Phage display was used to identify the most selective and specific ligands that can deliver nanocarriers to the tumor. The targeted liposomes, loaded with siRNA, were obtained by fusion of two types of liposomes: one containing siRNA and fusion phage protein (pVIII coat protein), and the other a MCF-7 cell-targeting peptide (DMPGTVLP). The presence of two fused proteins in the final liposomal formulation was further confirmed by Western blotting. Significantly downregulated PRDM14 gene expression followed by decreased PRDM14 protein synthesis in the target MCF-7 cells was also observed indicating that this approach has the potential for development as a new anticancer siRNA-based targeted nanomedicine [97]. The targeted liposomal system that encapsulated Lcn2 siRNA for selective targeting of MCF-7 and MDA-MB-231 cell lines was recently developed. The overexpression of Lcn2 in metastatic breast cancer promotes cancer progression by enhancing tumor angiogenesis and inducing epithelial-to-mesenchymal transition. The liposomes were PEGylated and decorated with octreotide peptide, which binds to somatostatin receptors overexpressed on breast cancer cells. The optimized liposomes had a mean size of 152 nm, a PDI of 0.13, a zeta potential of 4.10 mV, and high entrapment and loading efficiencies of 69.5% and 7.8%, respectively. In vitro studies showed that the OCT-targeted liposomes achieved approximately 55–60% silencing of Lcn2 mRNA. Additionally, the liposomes exhibited in vitro anti-angiogenic activity by reducing VEGF-A and HUVEC migration levels in the MCF-7 and MDA-MB-231 cells, suggesting their potential utility in inhibiting angiogenesis in MBC [98]. Liposomal drug delivery systems can also combine chemo- and immunotherapy as well as targeting as shown in Figure 3. Cationic liposomes conjugated with two DNA aptamers (called Aptm[DOX/IDO1]) to target cancer cells with the anticancer drug DOX and IDO1 siRNA, which reverses the immunosuppressive tumor microenvironment (TME). Aptm[DOX/IDO1] specifically delivered cargos to target sites via receptor-mediated endocytosis with aptamer-ligand binding. It demonstrated that the developed platform inhibits tumor metastasis by specifically targeting and efficiently delivering anticancer drugs to metastatic cancer cells via systemic circulation. Aptm[DOX/IDO1] effectively facilitated an immune response by inducing ICD and suppressing IDO1, promoting tumor regression in a subcutaneous breast cancer model mouse. The formulation has many advantages including site-specific drug delivery and accumulation in the targeted area, inhibition of PD-1/PD-L1 interaction, and inhibition of tumor metastasis by targeting circulating metastatic cancer cells [99]. To modulate the tumor immune microenvironment, pH-sensitive liposomes modified with cRGD and loaded with anemoside B4 (AB4) and programmed cell death ligand 1 (PD-L1) small interfering RNA (siP) were recently developed. This approach successfully co-delivered both AB4 and siP with good stability and targeted distribution in tumors, leading to improved immunosuppression and attenuation of tumor growth in two animal models. The results showed that cRGD surface modification of liposomes enhances cellular uptake and liposomes accumulation in the tumor tissue, followed by tumor growth inhibition against lung and breast cancer in vivo. Additionally, the co-delivery of AB4 and siP in AB4/siP-c-L resulted in the silencing of the PD-L1 gene and changes in the tumor microenvironment, which allowed the immune system to react better against cancer and induced long-term memory effects. This targeted nanovesicle approach has great potential for clinical application and offers a well-controlled design for investigating the effectiveness of combining herbal monomer components with various immunotherapies such as vaccines, cytokines, and antibodies [100].

### 3.2. Lipid Nanoparticles

The concept of electrostatic encapsulation of siRNA was also utilized in developing other nanocarriers, including cationic solid lipid nanoparticles (cSLN). The cSLN, based on 1,2-dioleoyl-sn-glycero-3-ethylphosphocholine, were prepared using emulsification solidification methods and characterized by paclitaxel and siRNA encapsulation efficiency. The use of cSLN increased the cellular uptake of fluorescently labeled dsRNA in human epithelial carcinoma (KB) cells. For the co-delivery of therapeutic siRNA, the human MCL1-specific siRNA (siMCL1) was complexed with PcSLN. In KB cells treated with siMCL1 complexed to PcSLN, MCL1 mRNA levels were significantly reduced and in vitro anticancer effects in KB cells were observed. Additionally, intratumoral injection of PcSLN complexed with siMCL1 significantly inhibited tumor growth in KB cell-xenografted mice [39]. 

Another promising lipid-based nanosystem for siRNA delivery to breast cancer includes LPNs. LPNs combine the advantages of both liposomes and polymers to achieve efficient siRNA delivery. The lipid bilayer can protect siRNA from degradation and enhance cellular uptake, while the polymer core can enhance siRNA encapsulation and stability. A recent study demonstrated the potential of LPNs for targeted siRNA delivery to breast cancer cells. Hybrid nanoparticles (HNPs) were created using a tri-block copolymer of PLA-PEG-PLA and DDAB cationic lipids. The method for preparing HNPs/siRNA complexes was simple and efficient, utilizing electrostatic interactions with lipids likely occurring at the interface of the hydrophilic shell and hydrophobic core. The HNPs demonstrated excellent biocompatibility, were easily internalized by MCF7 cells, successfully delivered siRNA into cells, and significantly and specifically downregulated the targeted IGF-1R gene [101]. Thus, these HNPs acting as siRNA delivery vehicles led to successful in vitro gene silencing and hold potential for use as effective nanomaterials for siRNA delivery with potential for reduced side effects in vivo. Recently, hybrid nanoparticles composed of phospholipids and PAMAM dendrimers were also developed to reverse multidrug resistance in cancer. The downregulation of P-glycoprotein (P-gp) using small interfering RNA (siRNA) was observed in breast (MDA-MB-231 and MCF-7) cancer cell lines. Nanoparticles containing generation 4 (G4) polyamidoamine (PAMAM)-PEG2k-DOPE and PEG5k-DOPE were surface modified with monoclonal antibody 2C5 (mAb 2C5) to target cancer cells. This active targeting of tumors results in increased drug and siRNA accumulation at the tumor site, thus minimizing off-target effects. In vitro studies have shown that the micelles have a higher cellular association and effectiveness [102,103]. Another targeted siRNA therapy for triple-negative breast cancer was developed using cationic lipid-assisted poly(ethylene glycol)-b-poly(d,l-lactide) (PEG-PLA) nanoparticles as the carrier. The study showed that only in TNBC cells that overexpress c-Myc, delivery of siRNA targeting cyclin-dependent kinase 1 (CDK1) with the carrier (NPsiCDK1), induced cell death through RNAi-mediated CDK1 expression inhibition, which indicates the synthetic lethality between c-Myc and CDK1 in TNBC cells. The NPsiCDK1 also suppressed tumor growth in mice bearing SUM149 and BT549 xenografts with no systemic toxicity or activation of the innate immune response, demonstrating the therapeutic potential of such nanoparticles loaded with siCDK1 for c-Myc overexpressed TNBC [64]. 

In summary, lipid-based nanosystems are a promising approach for siRNA delivery to breast cancer. These nanosystems can encapsulate siRNA, protect it from degradation, and deliver it to cancer cells. Nowadays, research on siRNA delivery to cancer focuses on combining multiple strategies to utilize the advantages of different delivery systems and to thus minimize the off-target effect. However further research is needed to optimize these lipid-based nanosystems for clinical translation.

## 4. Lipid-Based Nanosystems in Clinical Development for siRNA Drug Delivery to Breast Cancer

Lipid-based NPs have demonstrated promising outcomes in clinic and clinical trials [104,105,106]. siRNA-based drug delivery strategies signify an avenue for more effective and less toxic cancer therapy compared to chemotherapy. siRNA drugs may be applied in combination with standard therapy approaches to create a personalized and effective therapy result for breast cancer patients [107]. siRNA drugs could be utilized integrated with chemotherapy or immunotherapy to minimize systemic toxicity to improve the efficacy of siRNAs targeting resistance pathways in tumor cells [20,68,108]. 

The phase I trial investigated the best dose and side effects of EphA2-targeted DOPC (1,2-dioleoyl-sn-glycero-3-phosphatidylcholine) encapsulated siRNA (EphA2 siRNA for the treatment of patients with metastatic, advanced, and recurrent solid tumors. EphA2 siRNA reduced tumor growth by downregulating the gene that causes tumor growth. EphA2 siRNA was delivered using neutral liposomes. By intravenous injection in patients with advanced, metastatic, recurrent solid tumors, it may be applied for breast cancer treatment [108,109].

In 2014, a liposomal siRNA drug Atu027 was produced by Silence Therapeutics GmbH, targeting protein kinase 3. This siRNA drug was evaluated in a dose-escalation phase I clinical trial and resulted in disease stabilization in 41% of patients [110].

A lipid-based drug with two siRNAs targeting both VEGF and kinase spindle protein (KSP) (known as ALN-VSP) was tested on 41 patients with solid tumors in a phase I study. From a safety viewpoint, ALN-VSP was generally well tolerated and favorable. The result was promising with most of the patients having stabilized disease for approximately 8–12 months [111].

Another lipid-based siRNA drug called EnCore lipid NPs (DCR-PHXC-101) has been developed. DCR-PHXC-101 downregulates the expression of the transcription factor Myc, which is overexpressed in many solid tumors including breast cancer in a Phase 1 dose-escalation study [112]. Several lipid-based siRNA drugs in different phases of clinical trials for the treatment of solid tumors may be applied for breast cancer therapy (Table 3) [20,108].

For clinical evaluation of siRNA-based therapy, clinical trials must monitor and provide data on RNA-NP pharmacokinetics/pharmacodynamics, dosage and dose frequency, administration route, immunogenicity, bioavailability, biodistribution, biodegradation, and elimination pathway for FDA review as well as the physiochemical properties of the RNA-NP, including charge, size, and how it reacts to environmental factors such as pH, salt concentration, and temperature. In addition, the manufacturing processes and controls, such as stability, sensitivity, and purity/quality of the RNA-NPs during production and storage, must be assessed. The RNA-NP’s safety and efficacy depend on the targeted disease, but the treatment should have a significant therapeutic effect with minimal adverse side effects. Due to the extensive preclinical and clinical assessments required, along with the variation in acceptable efficacy and safety parameters, the number of FDA-approved RNA-NPs is currently limited [119,120,121].

Despite these challenges, three RNA-NPs have successfully met these requirements and gained FDA approval for public use in the United States. One of these is Onpattro^®^ (or patisiran), developed by Alnylam Pharmaceuticals Inc., which treats polyneuropathy associated with autosomal dominant hereditary transthyretin amyloidosis (hATTR). Onpattro^®^ consists of liposome-encapsulated siRNA that inhibits the expression of transthyretin (TTR), a protein released by the liver to transport thyroid hormone, which can undergo mutations causing hATTR [122]. Onpattro^®^ liposomes contain DLin-MC3-DMA (MC3), cholesterol, 1,2-distearoyl-sn-glycero-3-phosphocholine (DSPC), and PEG, with a diameter range of 60–100 nm [123]. After intravenous (IV) administration, Onpattro^®^ NPs localize in the liver via the RES, where they release siRNA in hepatocytes to inhibit TTR production and secretion. A crucial factor contributing to Onpattro’s^®^ success is the incorporation of the pH-sensitive ionizable lipid MC3. As the pKa of MC3 is ~6.5, matching the pH of the late endosome/lysosome, the liposome can fuse to the endosomal membrane after uptake and release therapeutic siRNA into the cytoplasm [124]. However, the need for IV infusion every three weeks limits the ease and widespread use of this RNA-NP therapy, even though it can suppress TTR expression by >80% [121,124].

While a few RNA-NPs have demonstrated success in clinical settings, most RNA-NPs do not progress through or complete clinical trials due to safety concerns and toxicity observed in both non-human primates and humans. To address these challenges and enhance the successful translation of RNA-NPs into clinical applications, there is a need for reliable methods to effectively and stably incorporate RNA, ensuring a low surface charge post-incorporation. Additionally, RNA-NPs should possess high stability, have a diameter of less than 100 nm to facilitate optimal cellular uptake, and be manufactured through established, large-scale, sterile processes [27].

## 5. Conclusions and Future Perspective

Breast cancer therapy utilizing lipid-based nanosystems significantly improves the delivery of siRNA to tumor cells. This approach has important advantages such as low therapeutic doses, reduced cytotoxicity to healthy cells, and inhibition of resistance to chemotherapy. Lipid-based nanosystems delivering siRNA to tumor cells can regulate the expression levels of genes associated with cell growth, proliferation, cell death, invasion, and metastasis. Fabrication of novel lipid-based siRNA nanosystems and novel targeting ligands may enhance breast cancer therapy as well as treatments for other diseases [125].

Recently, four siRNA drugs (patisiran, givosiran, lumasiran, and inclisiran) have received FDA approval for various diseases. siRNA drugs are in various phases of clinical trials based on different delivery systems and have been used to cover a wide range of pathologies including breast cancer [126].

Lipid nanosystems have enhanced the therapeutic potential of siRNA in several cancers including breast cancer. LNPs such as liposomes SLN, NLS, and NEs are suitable for incorporation of the siRNA. LNPs have efficiently delivered the siRNA drugs by stabilizing nucleic acid, extending the use of siRNA for numerous applications, and enhancing therapy for breast cancer. For further improvement in bioavailability, enhanced circulation in the blood, delivery to the target organ, surface functionalization of LNPs such as PEGylation, and introduction of surface ligands on the LNPs have all been effective approaches. Engineered strategies to ‘shed’ the incorporated targeting/functional moieties may improve the therapeutic effects once the carrier reaches its target [18].

Bone marrow deposition of LNPs utilized to treat solid tumors may prompt immune activation that could contribute to the antitumor effect and hinder the biodistribution of LNPs to tumors and reduce the therapeutic effect from immune activation [18].

siRNA-loaded NPs have shown promise due to their potential for gene silencing effects in cancer therapy. However, monitoring of in vivo release and distribution of siRNA is still challenging. To improve monitoring, the use of different imaging techniques, such as fluorescence imaging and CT imaging, may be an effective approach [127].

The LNP consisting of ionizable lipid, helper lipid, cholesterol, and PEG-encored lipid can stably encapsulate siRNA and improve release into the cytoplasm. Recently, noteworthy advancements were made in LNP research starting from the modification of LNP constituents, their formulation, and their application in breast cancer therapy to create a desirable composition and size, with a high siRNA encapsulation efficiency and a saleable product [128].

Lipid-based nanosystems are a promising strategy for siRNA delivery to breast tumors. These nanosystems can encapsulate siRNA efficiently, shield it from degradation, and deliver it effectively to cancer cells. Research focusing on siRNA delivery to breast tumors in a combination of multiple modalities to utilize the advantages of different delivery systems minimizing the off-target effects can combat breast cancer to save lives by reducing mortality rates.

Lipid-based nanocarriers such as SLNPs, liposomes, NLCs, and conjugates may be developed specifically for enhanced siRNA delivery and improved in vivo efficacy. Lipid-based NPs utilized for therapeutic approaches for siRNA delivery may be effectively used for targeting specific genetic elements of breast and other cancer cells leading to precise and efficient killing of cancer cells to inhibit gene expression or translation of cancer-allied proteins, reducing cell proliferation and tumor growth. This approach may improve greatly the stability and circulation time of siRNA molecules in the body, permitting more efficient therapeutic targeting of cancer cells with a potential to regulate the tumor microenvironment with decreased tumor immunosuppression.

## Figures and Tables

**Table 1:** Examples of lipid nanocarriers used for delivery of siRNA.

Lipid Nanocarrier Type	Lipids Used	Active Moiety Delivered	Key Conclusions	References
Lipid Nanoparticles (LNPs)	Ionizable cationic lipids (e.g., DOTAP, DLin-MC3-DMA) and helper lipids (e.g., cholesterol, PEG-lipids)	siRNA (Small interfering RNA)	LNPs effectively encapsulated siRNA and protected it from degradation. The formulation demonstrated high stability, efficient cellular uptake, and endosomal escape. LNPs efficiently delivered siRNA to target cells and achieved significant gene silencing, offering promising potential for therapeutic applications.	[18,19]
Solid Lipid Nanoparticles (SLNs)	Solid lipids (e.g., stearic acid, glyceryl monostearate) and surfactants (e.g., Tween, Span)	siRNA	SLNs provided a stable and biocompatible platform for siRNA delivery. The formulation protected siRNA from enzymatic degradation and facilitated cellular uptake. SLNs demonstrated effective gene silencing in vitro and in vivo, highlighting their potential as siRNA delivery systems.	[20]
Liposomes	Cationic lipids (e.g., DOTAP, DODAB) and neutral lipids (e.g., phosphatidylcholine)	siRNA	Liposomes efficiently encapsulated siRNA and protected it from nuclease degradation. The cationic lipids facilitated cellular uptake and endosomal escape of siRNA. Liposomal siRNA delivery showed effective gene silencing in target cells, making liposomes a promising option for siRNA therapeutics.	[21,22,23]
Cationic Lipid-DNA Complexes (lipoplexes)	Cationic lipids (e.g., Lipofectamine, Polyethylenimine) and plasmid DNA	siRNA or gene encoding siRNA	Cationic lipids formed stable complexes with siRNA or plasmid DNA and facilitated their cellular uptake. Lipoplexes efficiently delivered siRNA or gene encoding siRNA, resulting in effective gene silencing or knockdown of the target gene. Lipoplexes showed potential for siRNA-based therapeutics and gene therapy applications.	[12,24]
Ethosomes	Phospholipids (e.g., phosphatidylcholine) and ethanol	siRNA	Ethosomes provided enhanced permeation of siRNA through the skin or mucosal barriers. The formulation improved siRNA stability and promoted efficient delivery into target cells. Ethosomes showed potential for transdermal or mucosal siRNA delivery, offering opportunities for local or systemic treatments.	[25]

**Table 2:** Examples of lipids commonly used in delivery systems for siRNA [20,69].

Group of Lipids/Lipidoids	Examples
Cationic lipids	DOTAP, DOTMA, DC-6-14
Ionizable lipids/lipidoids	A6, A18-Iso-5-2DC18, 98N_12_-5, DLin-MC3-DMA, Lin-DMA, DODAP, DLin-KC2-DMA, DLin-MC3-DMA, YSK05, YSK13, CL4H6.
Sterols	Cholesterol, DC-cholesterol, Sitosterol
PEG-lipid conjugates	DSPE-PEG, DMG-PEG
Phospholipids	DOPE, DSPC, DSPC

**Table 3:** Lipid-based siRNA drugs are in different phases of clinical trials for the treatment of solid tumors.

siRNA Drug	Delivery System	Target	Cancer Type	Phase, Status	Company	NCT	Ref.
siRNA-EphA2	Liposomes	EphA2	Advanced Solid tumors	I, Active, Not Recruiting	M.D. Anderson Cancer Center	01591356	[113]
DCR-PHXC-101	Lipid-based NPs	Myc	Solid tumors	Terminated	Dicerna Pharmaceuticals	02110563	[114]
Atu027	Liposomes	Protein kinase 3	Solid tumors	I, Completed	Silence Therapeutics GmbH	00938574	[110]
ALN-VSP	Lipid-based NPs	VEGF, KSP	Solid tumors	I, completed Completed	Alnylam Pharmaceuticals	00882180 01158079	[115,116]
TKM-080301	Lipid NPs	PLK1	Advanced Solid tumors	II, completed	Arbutus Biopharma Corporation	01262235 02191878	[117,118]

## Data Availability

Not applicable.
